# Influencing Factors of Surfactant Stripping Crude Oil and Spontaneous Imbibition Mechanism of Surfactants in a Tight Reservoir

**DOI:** 10.1021/acsomega.2c02190

**Published:** 2022-05-26

**Authors:** Guangsheng Cao, Qingchao Cheng, Ying Liu, Ruixuan Bu, Ning Zhang, Peilun Wang

**Affiliations:** † Key Laboratory of Enhanced Oil & Gas Recovery of Ministry of Education, Northeast Petroleum University, Daqing 163318, P. R. China; ‡ No. 2 Oil Production Plant, Daqing Oilfield Co., Ltd., Daqing 163000, China; § Center of Chemistry for Frontier Technologies, Zhejiang University, Zhejiang 310028, China

## Abstract

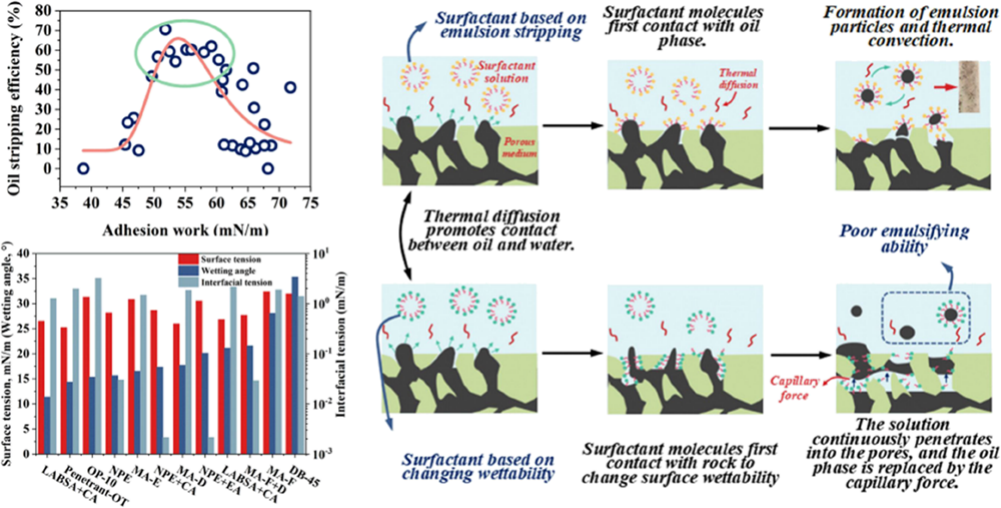

Surfactants play a vital role in the working fluid during the exploitation of tight reservoirs. The main goal is to clarify the mechanism of surfactant production enhancement in the reservoir. In this paper, starting from the interface properties and emulsifying properties of surfactants, the factors affecting the stripping of crude oil by different surfactants were described in detail. Meanwhile, the imbibition experiments of cores were used to clarify the two spontaneous imbibition mechanisms of the surfactant. Namely, they are the capillary force expulsion caused by the emulsion stripping thermal diffusion–convection and the wettability change. When the interfacial tension between the surfactant and oil is in the range of 10^–2^–10^–3^ mN/m, the particle size of emulsion is less than 1 μm, and the oil stripping efficiency is greater than 58%. The imbibition is mainly caused by thermal diffusion–convection. The wetting angle of the surfactant mainly changing wettability is less than 15°, and the adhesion work is greater than 52 mN/m. Using X-ray computed tomography, the surfactant imbibition distance of different permeability types of cores was obtained. The results show that higher permeability cores have a deeper imbibition distance. The results of this paper enrich the mechanism of enhanced oil recovery by surfactants and have important implications for the exploitation of tight reservoirs.

## 1 Introduction

The use of surfactants is considered to be an important method for the effective development of tight reservoirs.^1,2^ For the exploitation of tight reservoirs, large-scale volume fracturing is usually used to form fracture networks for huff-n-puff production.^3^ The surfactant is used as a constituent of fracturing fluid, which has two functions in the fracturing process.^4^ The fracturing fluid without flow back causes the liquid to remain in the dense porous medium. The pores of the fracture surface in the reservoir cause blockage and reduce the relative permeability of hydrocarbons. The surfactant can solve the blockage of the fracturing fluid. This is for the reason that a tight reservoir shows high capillary pressure, which captures fracturing fluid for a long time.^5^ However, the surfactant greatly reduces the interfacial tension between the fracturing fluid and the crude oil, weakens the capillary forces, and eliminates the blocking of the fracturing fluid.^6,7^ Additionally, in the course of the fracturing process, the surfactant makes mechanical shear action with the oil to form the emulsion under the action of displacement pressure in the reservoir. In the process of well huff-n-puff production, the surfactant plays a stripping role, forming emulsion to improve the recovery.^8,9^ The combination of the two effects can contribute to the development of tight reservoir fracturing stimulation. A surfactant with high oil stripping efficiency plays a vital role in the exploitation of tight reservoirs. Nevertheless, the influencing factors and stripping mechanism of the surfactant in the process of oil stripping on rock particles are less systematically expounded by scholars. Therefore, this paper elaborated on the influencing factors of oil stripping efficiency. Spontaneous imbibition also plays a crucial role in the fracturing process containing the surfactant. Spontaneous imbibition refers to the invasive process of a wetting phase displacing a non-wetting phase by means of capillary and/or gravity forces.^10,11^ Studies have shown that spontaneous imbibition is an effective tool for enhanced oil recovery.^11^ Several laboratory tests and field trials have shown that surfactant-enhanced imbibition can achieve promising results after hydraulic fracturing in unconventional resource exploration.^12−16^ At present, a lot of research work related to the imbibition of surfactant solutions has been carried out by related scholars.^17−20^ To name only a few, in the process of imbibition of anionic surfactant solution, the effects of capillary radius and surfactant solution properties on the position of the oil–water interface in oil wet horizontal capillary were studied.^21^ Sheng used a simulation method combined with theoretical analysis and published experimental results to study the spontaneous imbibition mechanism of a surfactant in the core scale.^22^ Liu and Sheng studied the effect of surfactant addition on shale self-permeability by nuclear magnetic resonance. They also tried to study the mechanism of anionic and nonionic surfactants on the wettability change of shale and analyze the influence of wettability change and interfacial tension reduction on the spontaneous imbibition process of oil-wet shale samples.^23^

The oil stripping efficiency of the surfactant and the mechanism of surfactant imbibition in porous media need to be fully described during the development of tight reservoirs. Few scholars have systematically expounded the influencing factors and mechanism of surfactants on oil stripping. For the imbibition process, the current research is mostly from the perspective of changing single factors such as wettability or interfacial tension, and there is a lack of multi-dimensional description of the imbibition mechanism. In this paper, on the basis of considering the factors affecting the stripping efficiency of surfactants, the mechanism of spontaneous imbibition in tight reservoirs is studied from the aspects of interfacial tension, wetting angle, emulsion stability, emulsion particle size, and imbibition distance. The research results of this paper are of great significance to their development in a tight reservoir.

## 2 Experimental Section

### 2.1 Materials and Reagents

The material used in this study includes 20 mesh quartz sand, crude oil (provided by No. 9 Oil Production Plant of Daqing Oilfield), natural cores (two physical properties), deionized water, and surfactants. The surfactants include 30% coconut fatty acid potassium (CPS), 90% sodium dodecyl benzene sulfonate (SDBS), 45% sodium diethylhexyl sulfosuccinate (Penetrant-OT), 99% alkylphenol polyoxyethylene ether (OP-10), 70% sodium lauryl ether sulfate (SLES), 90% nonylphenol ethoxylates (NPE), 92% sodium alpha-olefin sulfonate (AOS), 60% sodium diisobutylnaphthalene sulfonate (Penetrant-BX), 60% secondary alkane sulfonate sodium (SAS), 60% sodium dodecyl sulfate (SDS), 35% cocoamidopropyl betaine (CAB), 50% sodium alcohol ether sulfate (AES), 30% potassium laurate soap (SLP), 80% polysorbate (T-80), 30% dodecyl dimethyl betaine (BS-12), 35% lauramidopropyl hydroxy sulfobetaine (LHSB), 35% lauramidopropyl betaine (LAB), 45% sodium dodecyl diphenyl ether disulfonate (DB-45) produced by Qingdao USOLF company, China, and 70% ammonium laureth sulfate (ALES) produced by Shandong LUSHEN company, China. The concentrations of surfactant used in the experiment were all 0.5%. In order to increase the experimental samples, the mixed agents of dodecylbenzenesulfonic acid (LABSA) + AES, LABSA+coconut oil fatty acid (CA), MA-D, MA-E, MA-F, MA-G, MA-H, MA-I, and MA-J produced by Daqing Oilfield Industrial Co., Ltd. were used with the existing compounding agents. The experimental concentration of the abovementioned single mixed solvent was 0.5%, and 0.25% was used for each mixed agent.

The mixed agent of 0.45% sodium linear-dodecylbenzenesulfonate (LAS) + 0.35% coconut diethanol amide (CDEA) + 0.25% ethyl alcohol (EA) was also prepared by compounding the surfactant monomer produced by Qingdao USOLF Company.

The interfacial tension and wetting angle were measured by a model TX-500D rotating drop interfacial tension meter and model A801S dynamic and static contact angle meter, Kino, USA, respectively.

### 2.2 Oil Stripping Efficiency Experiment

For tight reservoirs, the oil in the formation is adsorbed on the rock surface for a long time, which makes it difficult for the external fluid to enter the tight reservoir. The oil on the surface of porous media can be stripped by the surfactant entering the porous media. In order to clarify the influencing factors of oil stripping, experiments on the stripping of oil from quartz sand surface by different surfactants were carried out.

The experimental steps are as follows:

1) The washed quartz sand was mixed with crude oil in the ratio of 1:6 to make the oil sand, with the mixture aged for 24 h at 60 °C.
2) The surfactant solution with a mass concentration of 0.5% was mixed with deionized water.
3) The oil sand (*m*_1_ = 15 g) was placed in the centrifuge tube, and the surfactant was added according to the mass ratio of oil sand:surfactant = 1:2. The samples were put into the centrifuge, which can make the oil sand more compact and achieve the effect of the stripping static test.
4) The mixture in the centrifuge tube of step 3 was filtered, and the filtered oil sand was wrapped and put into a drying oven for 24 h.
5) The oil sand in step 4 was weighed, and the oil stripping efficiency was calculated using eq 1.

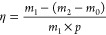
1where η is the oil stripping efficiency, %, *m*_0_ is the mass of the centrifuge tube, *g*, and *p* is oil content, %(*m*_1_ × *p* = 2.14*g*).

### 2.3 Emulsion Stability and Emulsion Size Measurement Experiment

Due to the influence of the surfactant, the stability and particle size of emulsion changed obviously. Therefore, the experiment of emulsion performance is divided into two parts, namely, the stability and emulsifying effect of emulsion are measured from macroscopic and microscopic perspectives.

#### 2.3.1 Measurement of Emulsion Particle Size under a Microscope

1) Oil and surfactant solution were mixed in a ratio of 1:1, 50 mL each, and placed in an incubator at 60 °C;
2) The solution was put into the emulsion mixer, the speed was set at 10,000 r/min, it was fully stirred, waiting for the emulsion to form;
3) A drop of emulsion was taken in step 2 and prepared on the worktable to adjust the microscopic light to make the display visible;
4) Appropriate magnification needed to be selected, and the pixel points of oil droplets were captured under the microscope to calculate the size of oil droplets.

#### 2.3.2 Experiment of Precipitation in a Colorimetric Tube

The 100 mL emulsion was put into the colorimetric tube and placed at 60 °C. The precipitation amount of oil and surfactant was read out and recorded at 0, 2, 4, 6, 10, 18, 30, 45, 60, 90, 120, and 180 min.

### 2.4 Spontaneous Imbibition of Oil and X-CT Scanning

**Figure 1:**
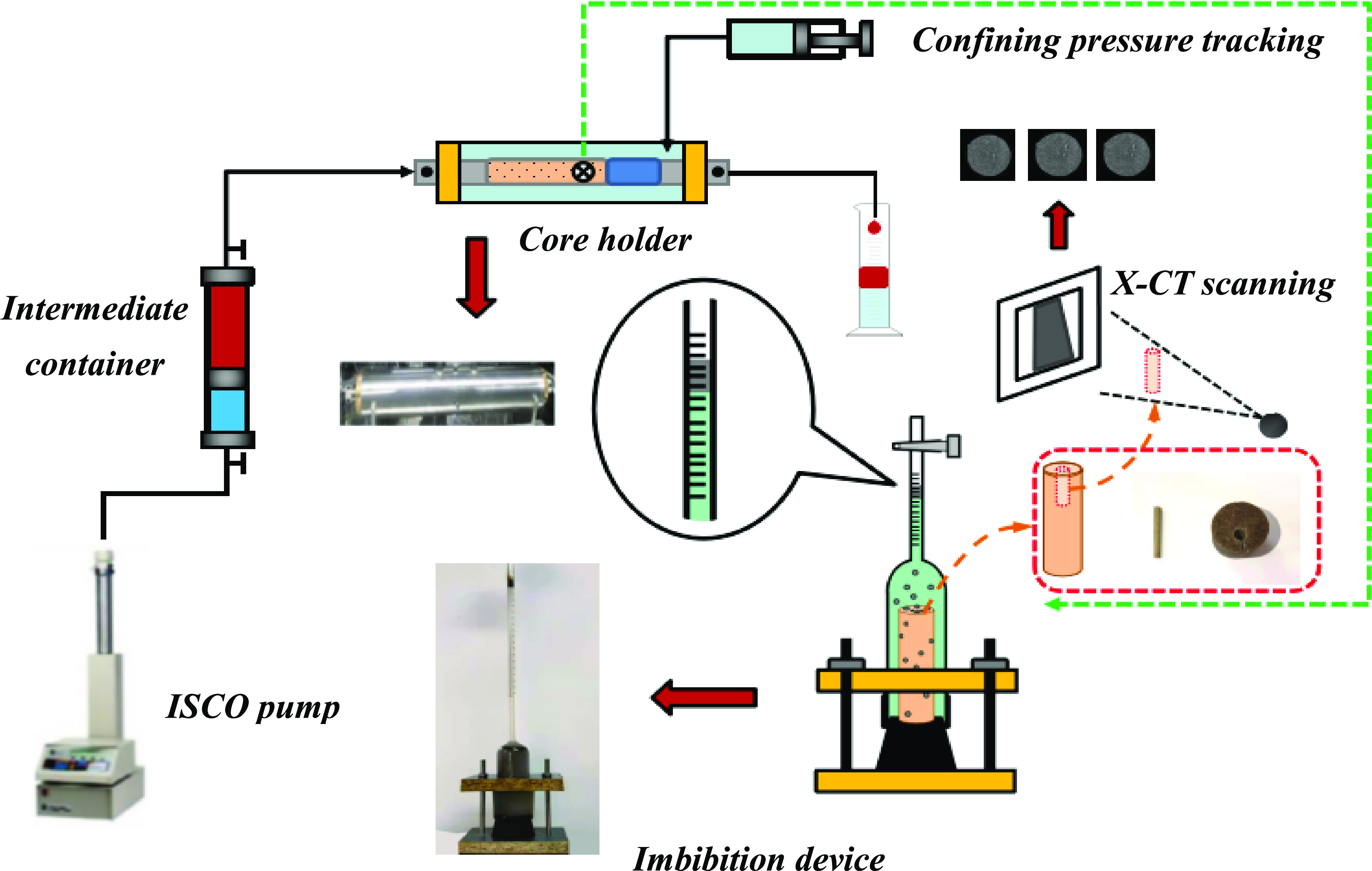
Spontaneous imbibition of crude oil and X-CT scanning.

The oil on the surface of the rock is stripped by the action of the surfactant and then enters the rock pore and moves freely. So as to analyze the spontaneous imbibition process of oil in porous media, the influence of free movement of emulsion in porous media on spontaneous imbibition was studied. Meanwhile, it is necessary to further derive the fluid distribution in porous media, and X-CT scanning of rock samples is needed (Figure 1).

1) Core was saturated with formation water, and the porosity was calculated;
2) The oil with the same viscosity under formation conditions was prepared (the formation of crude oil flowing to the ground and the increase of viscosity, kerosene was used for blending). The oil was saturated through the multi-functional core displacement device and aged for 24 h;
3) Core was placed in an imbibition flask containing different surfactants and the temperature was maintained at 60 °C;
4) The volume of oil in the imbibition flask at different times was measured;
5) The core after imbibition experiment was cut along the axis into a cylinder (3 mm in diameter × 30 mm in height) for X-CT scanning.

## 3 Results and Discussion

### 3.1 Effect of Surfactant Interface Properties on Oil Stripping Efficiency

The interfacial properties of different surfactants directly affect the stripping effect of oil from the surface of rock particles. From a macro perspective, the characterization of interfacial properties includes water–air surface tension, water–oil interfacial tension, and the wetting effect between gas–liquid–solid three phases. To clarify the stripping effect of surfactants on crude oil from the surface of quartz particles, it is necessary to measure the surface tension of different surfactants, the interfacial tension with oil, and the wetting angle on quartz flakes. The relationship between the interfacial properties of surfactants and oil stripping efficiency was obtained. The specific data are shown in Table 1.

**Table 1:** Experimental Results of Surfactant Parameters

surfactant name	surfactant type	surface tension (mN/m)	interfacial tension (mN/m)	wetting angle (°)	oil stripping efficiency (%)	adhesion work (mN/m)
CPS	anionic	22.781	2.74	6.40	12.15	45.42
SDBS	anionic	24	1.81	11.12	9.21	47.55
MA-G		23.616	8.16	11.16	25.7	46.79
LABSA + AES	anionic	26.506	1.27	11.38	59.35	52.49
MA-I		19.6	2.01	12.49	0.036	38.74
Penetrant-OT	anionic	25.225	1.98	14.33	46.73	49.67
OP-10	nonionic	31.32	3.24	15.34	50	61.52
SLES	anionic	31.835	3.19	15.34	11.68	62.54
NPE	nonionic	28.119	0.03	15.63	60.28	55.20
AOS	anionic	32.544	4.08	15.81	9.81	63.86
MA-E		30.805	1.48	16.50	55.14	60.34
NPE + EA		28.639	0.00214	17.31	61.35	55.98
MA-D		25.929	1.85	17.71	56.54	50.63
NPE + EA	nonionic	30.523	0.002153	20.10	61.91	59.19
Penetrant-BX	anionic	35.445	5.46	20.13	11.68	68.72
SAS	anionic	31.597	3.01	20.50	12.15	61.19
ALES	anionic	35.22	0.59	20.57	0	68.19
LABSA+CA		26.818	2.15	21.13	70.56	51.83
MA-E + MA-D		27.693	0.0289	21.59	54.21	53.44
SDS	anionic	33.532	3.49	22.00	8.88	64.62
CAB	amphoteric	34.522	2.2	23.20	10.28	66.25
AES	anionic	34.042	5.12	23.32	13.08	65.30
SLP	anionic	35.298	5.62	23.69	11.68	67.62
T-80	nonionic	37.686	0.13	25.15	41.12	71.80
BS-12	amphoteric	34.662	2.37	25.28	30.84	66.00
MA-F		32.398	1.89	28.07	44.86	60.99
LHSB	amphoteric	35.08	4.22	15.43	50.84	68.90
MA-H		32.621	1.02	30.12	39.02	60.84
LAS + CDEA		36.478	0.00169	31.28	22.35	67.65
MA-J		24.869	2.38	32.61	23.36	45.82
LAB	amphoteric	35.205	2.02	34.78	42.52	64.12
DB-45	anionic	31.935	1.4	35.31	58.88	58.00

**Figure 2:**
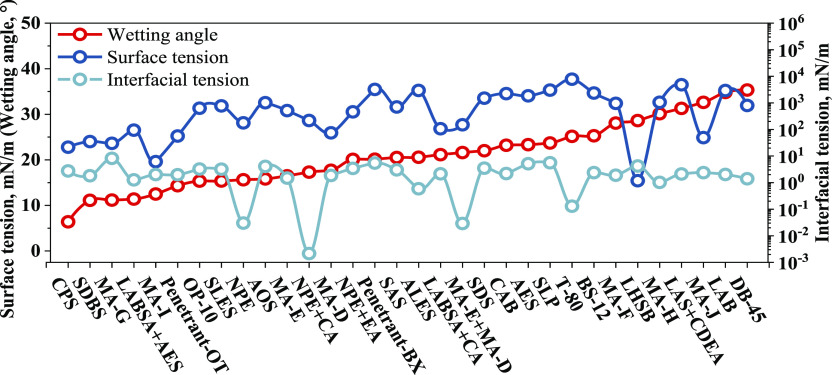
Interfacial property parameters of surfactants.

Figure 2 depicts the change of interfacial parameters of each surfactant. The wetting angles of different surfactants are sorted in an increasing manner, and it can be seen that the surface tension corresponding to the surfactant also increases. The wetting angle is determined by the surface tension and interfacial tension. For the same solid, the wetting angle is positively correlated with the surface tension. The corresponding surface tension and interfacial tension reflecting the interfacial energy between liquid–gas and liquid–liquid are consistent. Due to the difference in molecular properties of surfactants, the corresponding interfacial tension is reduced, resulting in differences.

**Figure 3:**
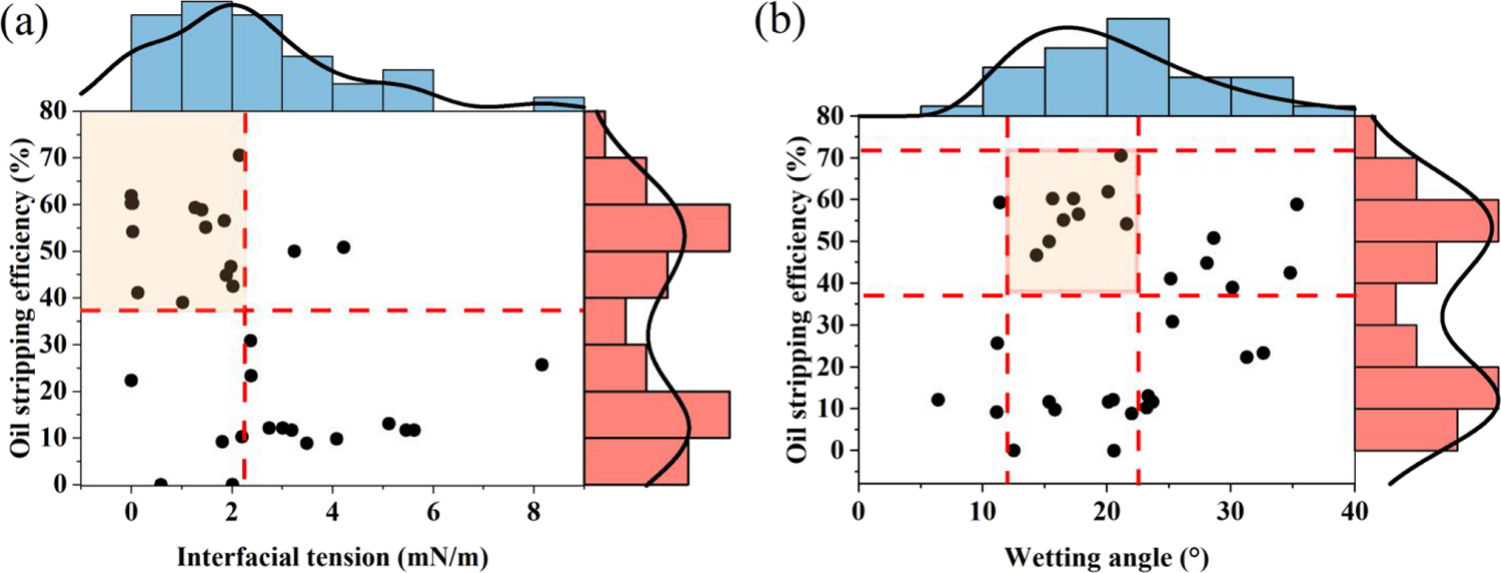
Boundary histogram of the relationship between interface properties and oil stripping effect: (a) interfacial tension vs oil stripping efficiency; (b) wetting angle vs oil stripping efficiency.

It can be seen from Figure 3 that the high frequency scatter points of interfacial tension and wetting angle corresponding to the oil stripping efficiency appear in the upper left and upper middle, respectively. Surfactants with interfacial tension below 2 mN/m and wetting angles between 10° and 22° can achieve high oil stripping efficiency. However, it is not accurate to judge the wettability only by the wetting angle. The wetting phenomenon is a process in which one fluid replaces another on the solid surface. From the thermodynamic point of view, the wetting degree is whether the surface free energy (adhesion work) of the system can be reduced (increased) under constant temperature and constant pressure.

Free energy (adhesion work) can be expressed by Young equation:
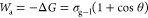
2where −Δ*G* is free energy, *W*_a_ is adhesion work, mN/m, σ_g–l_ is surface tension, mN/m, and θ is the wetting contact angle, °.

The adhesion work of each surfactant is calculated as shown in Table 1.

**Figure 4:**
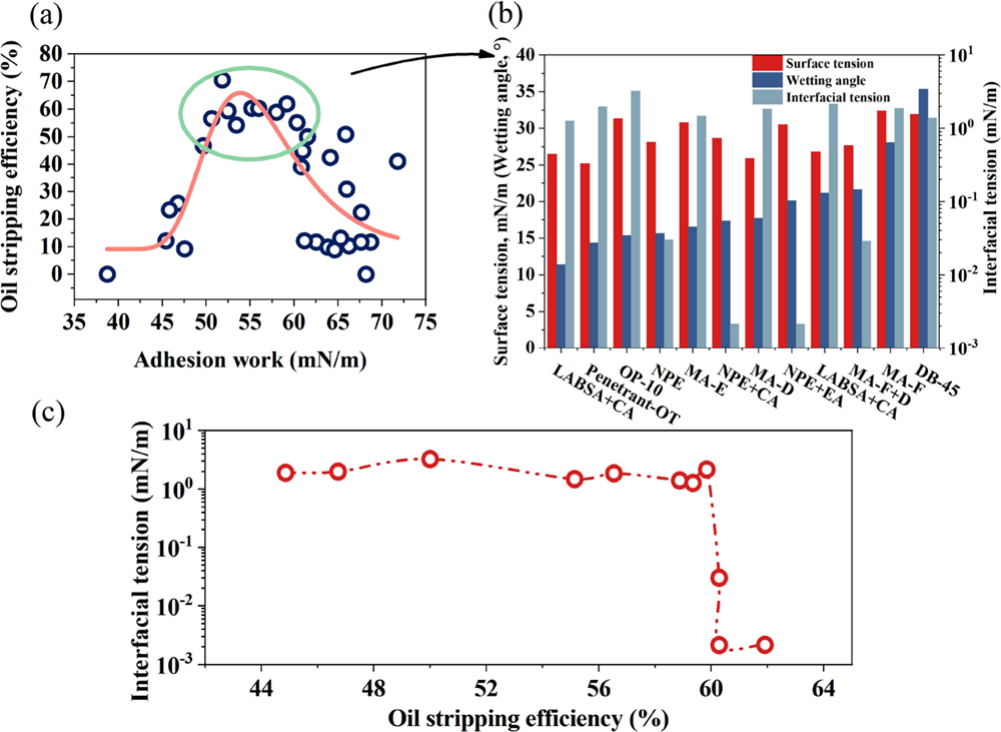
Relationship curve between adhesion work and oil stripping efficiency: (a) scatter diagram of adhesion work and stripping efficiency; (b) interfacial parameters corresponding to the adhesion work in the high oil stripping efficiency range; (c) interfacial tension changes of surfactants in the region of high oil stripping efficiency.

It can be found that the adhesion work has a high oil stripping efficiency within a certain range from Figure 4a. When the adhesion work is 49–62 mN/m, the corresponding stripping efficiency can reach 46.73–70.56%. As the adhesion work increases, the oil stripping efficiency has a tendency to increase and then decrease. This is the reason that high adhesion work makes most of the surfactant molecules adsorbed on the surface of quartz particles, reducing the adsorption on other particles. While there is a certain probability of adsorption of crude oil on the surface of sand particles, surfactants are mostly adsorbed on a single particle, reducing the stripping efficiency of the whole system. The surfactants with adhesion work in the range of 49–62 mN/m were screened. It is found that the interfacial tension of the surfactants is below 2 mN/m, the surface tension is 25–32 mN/m, and the wetting angle is between 11° and 35°, which can achieve high stripping efficiency. Figure 4c indicates that in the optimal adhesion work range, the interfacial tension of surfactants with high oil stripping efficiency decreases with the increase of stripping efficiency.

### 3.2 Effect of Emulsifying Property of Surfactant on Oil Stripping Efficiency

Emulsion is formed due to mechanical shearing action of porous media in the process of injecting surfactants into a reservoir. Similarly, some surfactant solutions can spontaneously form emulsions with oil under thermal convection. The blockage and retention of large emulsion particles in porous media directly affect the flow of fluid. Therefore, it is indispensable to study the effect of surfactant emulsification on crude oil stripping.

#### 3.2.1 Effect of the Demulsification Rate on Oil Stripping Efficiency

According to the experimental steps in Section 2.3, the stability and particle size of the emulsion formed by surfactant and oil were studied. First, the amount of water separated out from surfactant emulsion was measured and the demulsification rate was calculated.

**Figure 5:**
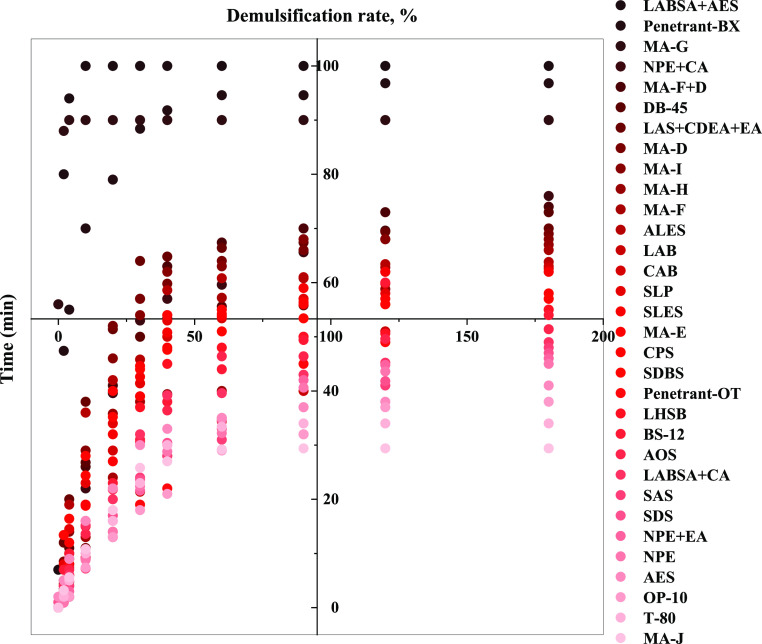
Precipitation and demulsification rate of emulsion at different times.

**Figure 6:**
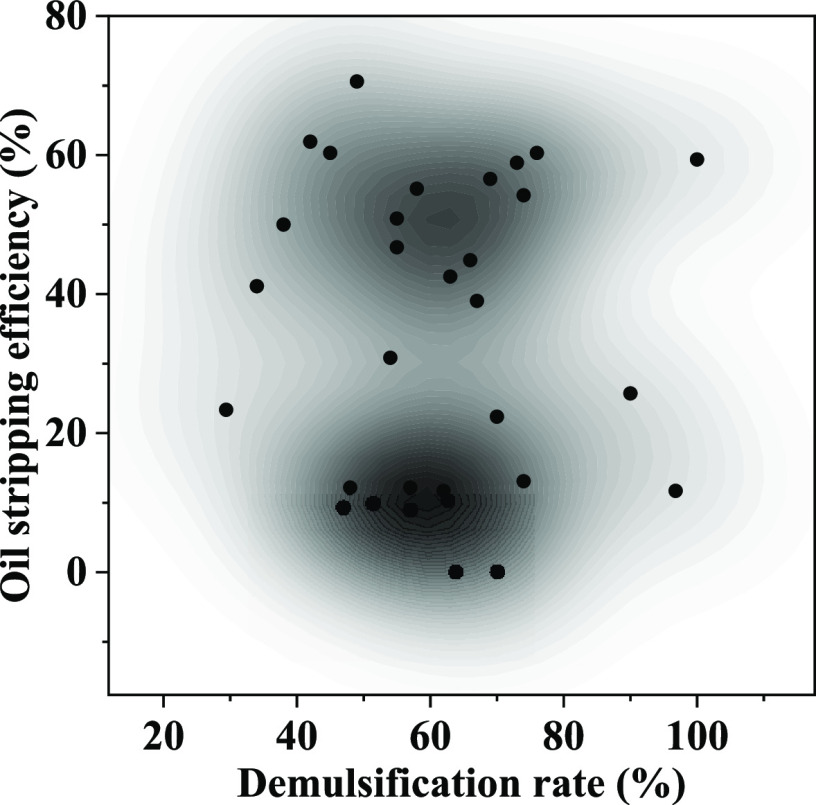
Density diagram of demulsification rate and stripping efficiency.

From the aqueous phase precipitation volume of the emulsion in Figure 5, it can be seen that most of the surfactants precipitated in the volume of 20–35 mL after standing for 180 min, and the demulsification rate was mainly concentrated above 30%. On the kernel density diagram of demulsification rate and stripping efficiency (Figure 6), there are two cores in the demulsification rate of 42–80%, corresponding to the stripping efficiency range of 0–20% and 40–60%, respectively, so there is no correlation between the demulsification rate and stripping efficiency.

#### 3.2.2 Effect of Emulsion Particle Size on Oil Stripping Efficiency

**Figure 7:**
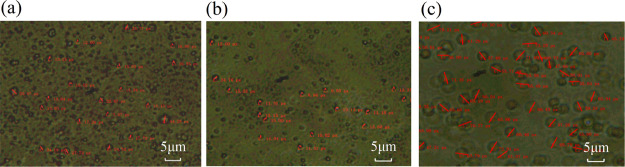
Emulsion particle size diagram: (a) LABSA + CA solution; (b) NPE solution; (c) SLP solution.

The emulsion was placed under a microscope for observation, and the pixels of the droplets under the microscope were recorded by selecting a suitable scale. The size of oil droplets was calculated by comparing the scale. The images of the emulsion particle size magnified 400 times are shown in Figure 7.

**Figure 8:**
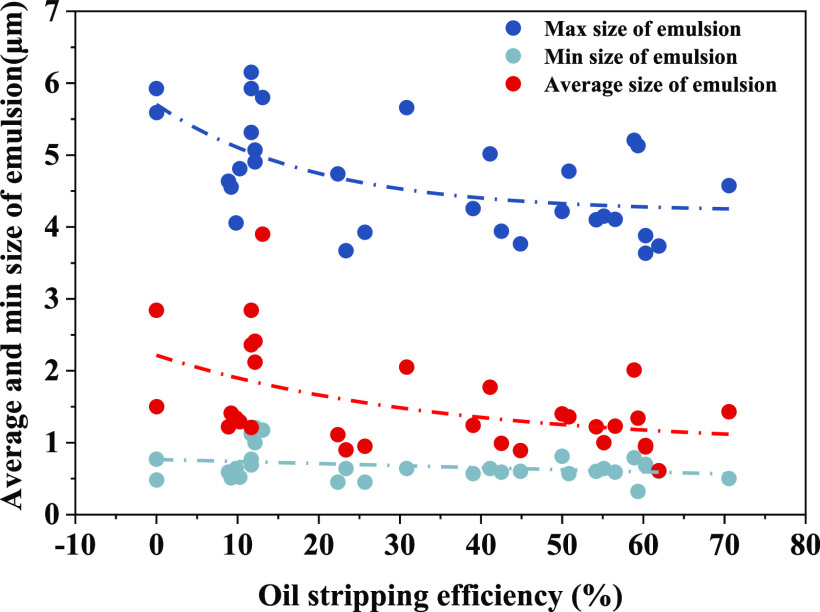
Relationship between oil stripping efficiency and emulsion particle size.

The size of emulsion particles in the picture was calculated to obtain the maximum, minimum, and average particle sizes of emulsion particles. The specific data are shown in Figure 8 and Table 2.

**Table 2:** Particle Sizes of Different Surfactant Emulsions

surfactant name	average size of emulsion (μm)	surfactant name	average size of emulsion (μm)
ALES	2.84	MA-H	1.24
MA-I	1.5	T-80	1.77
SDS	1.22	LAB	0.99
SDBS	1.41	MA-F	0.89
AOS	1.34	OP-10	1.4
CAB	1.29	LHSB	1.36
Penetrant-BX	1.21	MA-F + MA-D	1.22
SLES	2.84	MA-E	1
SLP	4.36	MA-D	1.23
CPS	2.41	DB-45	2.01
SAS	2.12	LABSA+AES	1.34
AES	3.9	MA-H + MA-B	0.96
LAS + CDEA+EA	1.11	NPE	0.94
MA-J	0.9	NPE + EA	0.61
MA-G	0.95	LABSA+CA	1.43
BS-12	2.05		

From the experimental results, the average size and maximum size of the emulsion are negatively correlated with the stripping efficiency, that is, the larger the emulsion size, the lower the stripping efficiency, but the minimum size has little relationship with the stripping efficiency. The minimum size of most emulsions is about 0.5 μm, and the average size is between 1 and 2 μm.

The oil stripping efficiency characterizes the ability of the surfactant to strip crude oil from the surface of sand particles. However, under formation conditions, the ability of the surfactant to strip the oil cannot be accurately described by simply evaluating the stripping efficiency. Therefore, it is necessary to further study the influence of the surfactant on the spontaneous imbibition of oil in porous media.

### 3.3 Effect of the Surfactant on Spontaneous Imbibition of Oil in a Tight Core

According to the experimental procedure described in Section 2.4, surfactants with oil stripping efficiency higher than 50% were selected, miscible solvents with unclear specific composition were removed, and T-80 with interfacial tension at the level of 10^–1^ mN/m and LAS + CDEA + EA at the level of 10^–3^ mN/m were added for imbibition experiments. The core physical parameters used in the experiment are adjusted to two tight reservoir levels according to reservoir classification standard. Before performing spontaneous imbibition experiments, the wettability of the cores was measured and cores with similar wettability were selected for the experiments. The basic parameters of the core are shown in Table 3.

**Table 3:** Core Basic Parameters

core number	surfactant name	oil stripping efficiency (%)	wetting angle (°)	core physical parameters			reservoir classification standard			
				gas permeability (10^–3^ μm^2^)	porosity(%)	oil saturation(%)	gas permeability (10^–3^ μm^2^)	porosity(%)	oil saturation(%)	reservoir type
C-1	OP-10	50	24.74	1.33	13.97	61.4	≥0.5	≥12	≥58	Type I
C-2	LHSB	50.84	25.17	1.5	14.28	63.5				
C-3	DB-45	58.88	24.96	1.36	13.83	62.6				
C-4	LABSA + AES	59.35	22.86	1.82	15.08	64.7				
C-5	NPE	60.28	24.13	1.42	13.86	61.8				
C-6	NPE + CA	61.35	23.21	1.57	13.72	59.8				
C-7	LABSA + CA	71.56	22.57	1.33	14.17	64.9				
C-8	T-80	41.12	21.93	1.25	13.95	60.3				
C-9	LAS + CDEA + EA	22.35	25.49	1.39	14.26	58.9				
C-10	OP-10		32.61	0.33	9.46	48.5	0.1 ∼ 0.5	8 ∼ 10	40 ∼ 50	Type II
C-11	LHSB		29.37	0.27	8.9	47.3				
C-12	DB-45		28.49	0.26	8.37	41.2				
C-13	LABSA + AES		31.57	0.23	10.28	45.1				
C-14	NPE		29.64	0.18	8.68	40.3				
C-15	NPE + CA		27.13	0.29	7.85	43.8				
C-16	LABSA + CA		30.89	0.18	8.41	49.6				
C-17	T-80		28.74	0.25	10.1	44.3				
C-18	LAS+ CDEA +EA		32.1	0.31	9.21	46.5				

**Figure 9:**
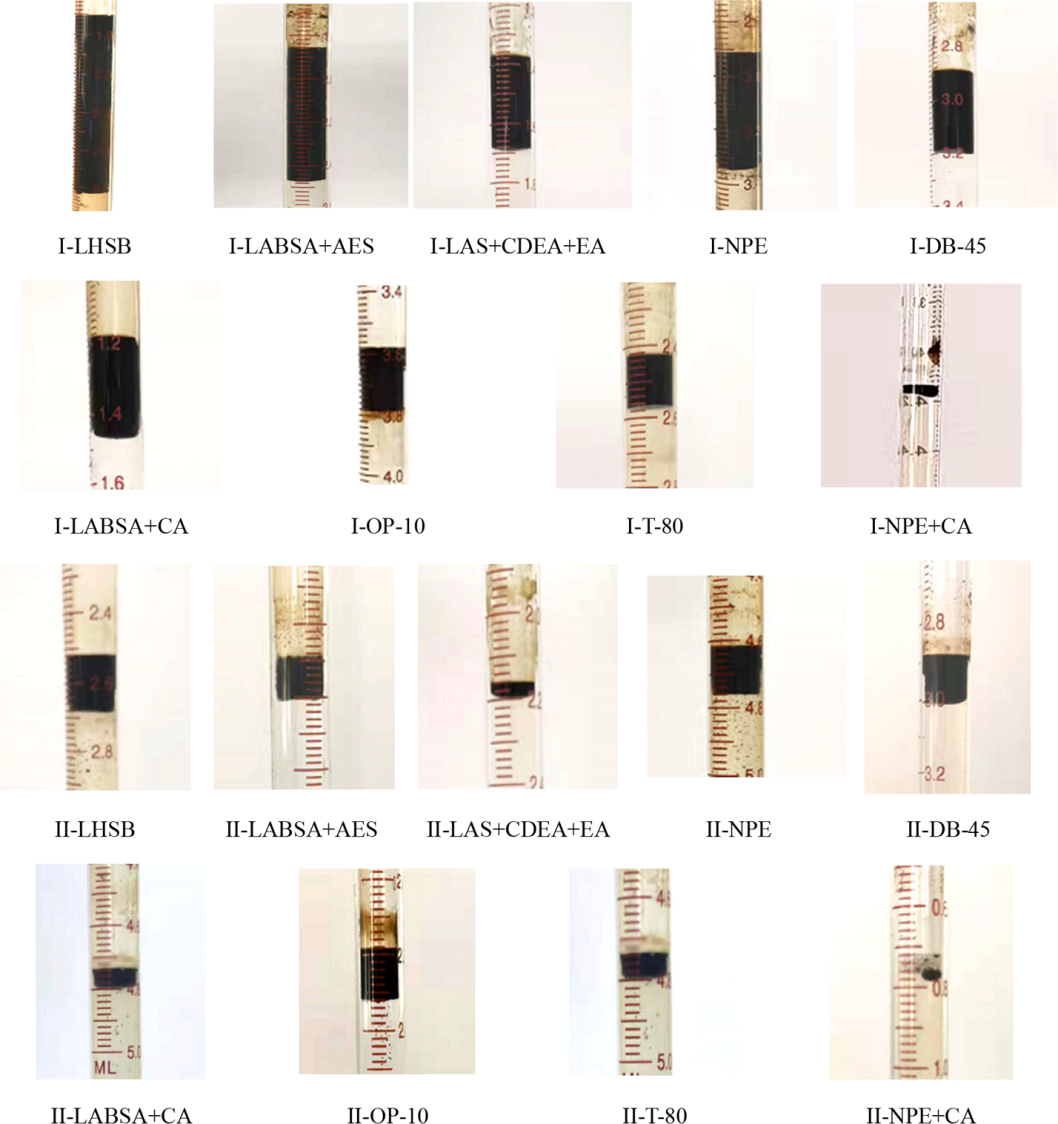
Imbibition amount of each surfactant under two tight reservoir types.

**Figure 10:**
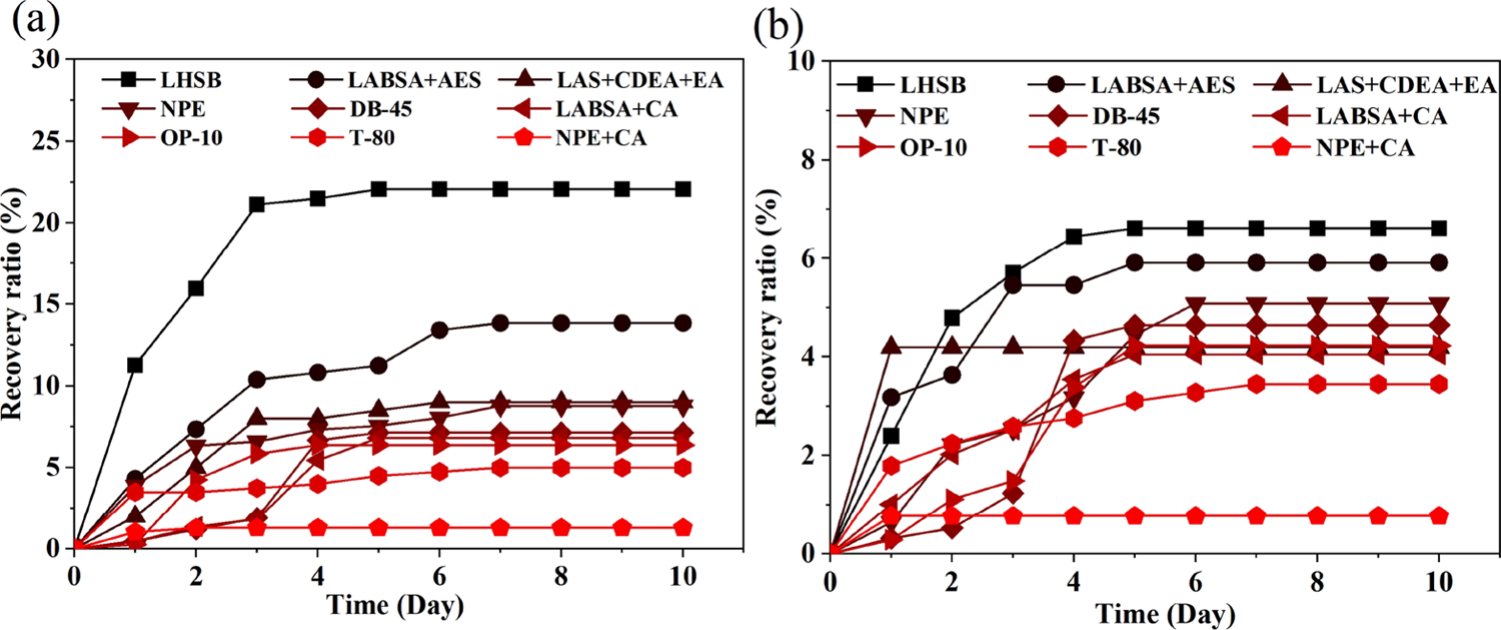
Variation curve of imbibition recovery rate: (a) imbibition recovery of the type I reservoir and (b) imbibition recovery of the type II reservoir.

The cumulative amount of imbibition at different times was recorded. The experimental results are shown in Figures 9 and 10.

It can be seen from Figure 10a,b that in the beginning of 0–4 days, the oil increased rapidly, and the increasing rate of the core with high permeability was higher. After 5 days, the increase of oil production slowed down, and the cumulative amount of oil was basically stable after 10 days. It indicates that the imbibition distance between the surfactant and oil is limited under the current conditions. It is difficult to obtain high imbibition recovery even if the surfactant has high oil stripping efficiency. Meanwhile, the imbibition recovery rates of the selected surfactants in type I reservoirs are 1–22%, mostly between 5 and 13%. The imbibition recovery of the type II reservoir is lower than that of the type I reservoir, which is distributed between 0.5 and 6%. The variation law of imbibition recovery with time of the both type reservoirs is consistent. Overall, the results point out that permeability is positively correlated with the imbibition recovery in the current permeability range. Figure 10b shows that at lower permeability reservoirs, the difference in recovery rates for each surfactant decreases. Combined with Tables 1, 2, and Figure 10, it can be seen that the imbibition recovery is not only related to the single property of surfactant, so it is necessary to further study the mechanism of surfactant imbibition recovery.

### 3.4 Spontaneous Imbibition Mechanism of a Surfactant in a Tight Reservoir

During the experiment, it was found that during the imbibition process of tight cores, the imbibition phenomena of surfactants in different systems were different, and the performance of surfactants in the imbibition process mainly included two aspects. First, surfactants that can achieve ultra-low interfacial tension are based on emulsion stripping and thermal diffusion–convection to achieve oil production, and the surfactant system is more turbid. The second is the surfactant, which can greatly change the wetting ability. The working fluid system is more clear, and the oil flows out from the void and adheres to the rock surface in the form of droplets. Although the interfacial tension of the surfactant is relatively large, the recovery rate is higher.

**Figure 11:**
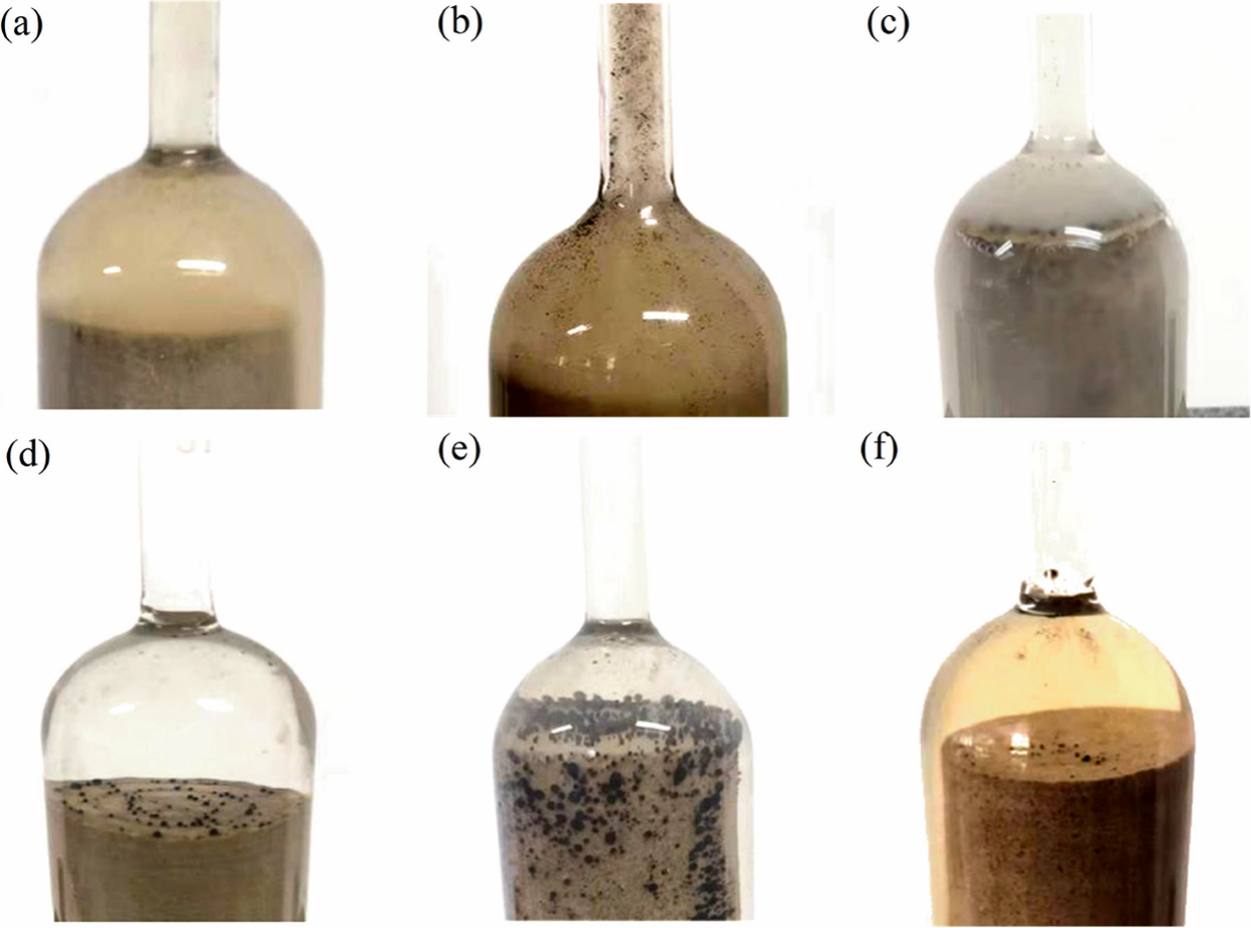
Imbibition phenomenon of different surfactants: (a) LABSA + CA solution; (b) NPE solution; (c) OP-10 solution; (d) LABSA + AES solution; (e) LHSB solution; and (f) LAS + CDEA+EA solution.

**Figure 12:**
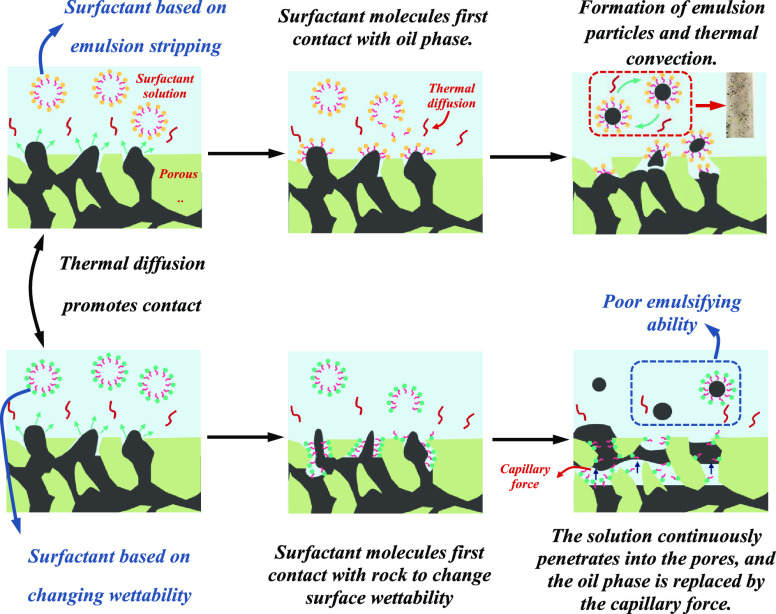
Schematic diagram of the permeation mechanism of two types of surfactants.

Figure 11a–c shows the first type of imbibition. Emulsification stripping and thermal diffusion–convection play a major role, while capillary force has little effect. This type of surfactant is mainly characterized by low interfacial tension (10^–2^–10^–3^ mN/m), small emulsion particles (less than 1 μm), and high oil stripping efficiency (more than 58%). Most surfactant molecules are combined with oil to form emulsion particles and can further contact with the oil in the pores through thermal convection so as to strip the oil from the porous medium. Figure 11d–f shows the second type of imbibition. Thermal diffusion promotes the contact of the surfactant with the rock surface and oil. However, the surfactant is easier to adsorb on rocks, which can greatly reduce the wettability of the core surface. With the continuous penetration of surfactant solution into porous media, capillary force displaces the oil phase and the surfactant and oil exhibit a displacement phenomenon. This type of surfactant has poor emulsifying ability and is not easy to form emulsion. The surfactant is characterized by a low wetting angle (less than 15°) and interfacial tension in the range of 10^0^ mN/m. The two types of imbibition mechanism are shown in Figure 12.

**Figure 13:**
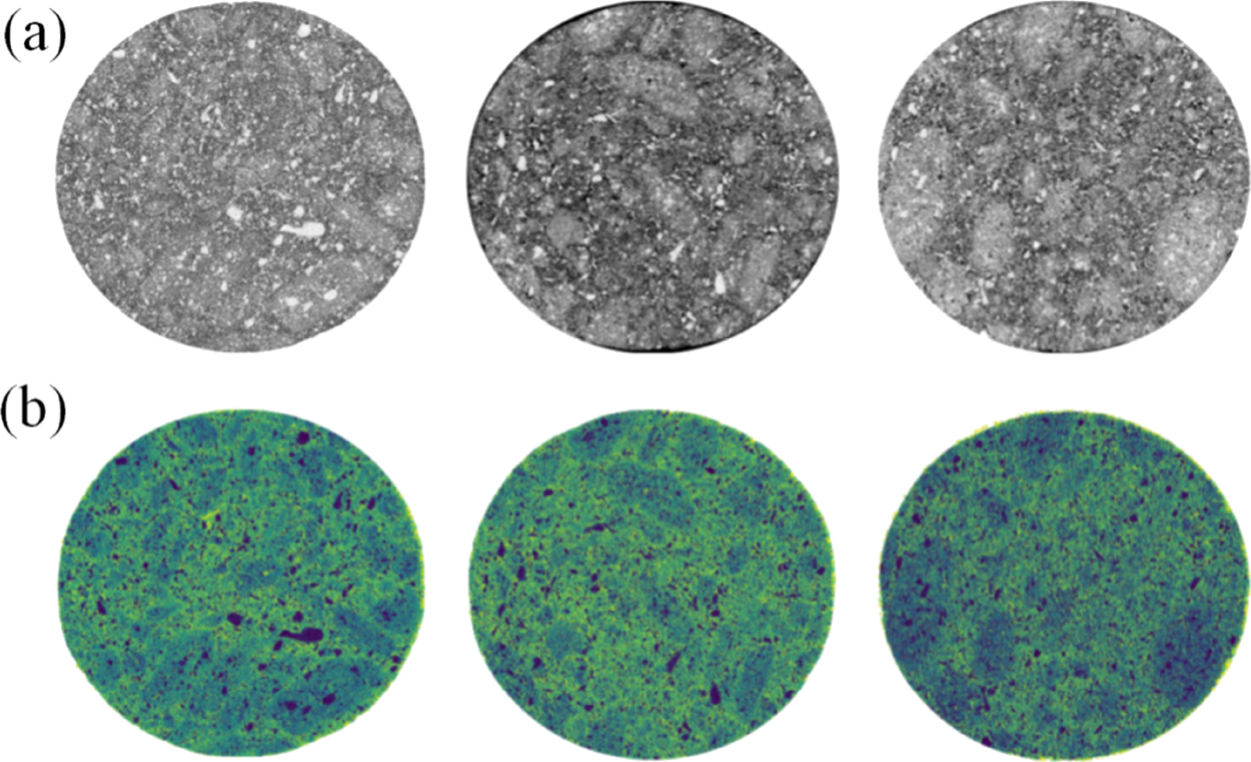
X-CT scanning pictures of cores at different positions: (a) X-CT scanning gray map of the type I reservoir and (b) X-CT scanning map after identification. The top, middle, and bottom of the core are shown from left to right.

**Figure 14:**
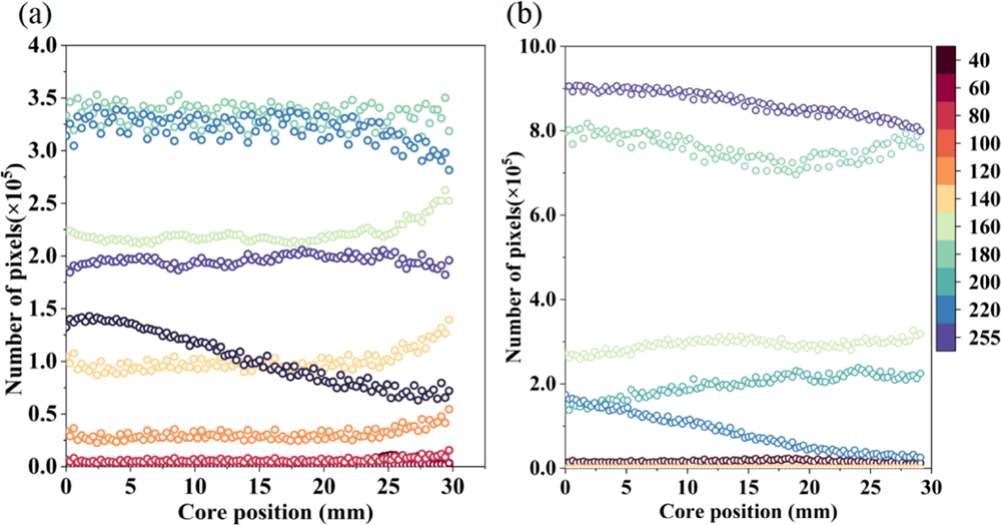
Gray distribution at different positions: (a) type I reservoir and (b) type II reservoir.

In order to further clarify the action distance of the surfactant and the remaining oil distribution after imbibition, X-CT scanning was performed on the core after imbibition to obtain the relative volume of fluid at different positions of the core. The NPE in type I and type II reservoir rock samples was selected for X-CT scanning because the NPE’s imbibition recovery was within the scope of most of the surfactant’s imbibition recovery. Since the CT scan is based on the relative density to determine the location of the fluid and rock skeleton distribution, it is transformed into a grayscale image. The gray range of fluid can be distinguished to determine the relative distribution volume of fluid. The X-CT scanning sample was taken out from the core after imbibition from top to bottom, and the distribution pattern of fluid-rock skeleton in each layer was swept uniformly. The number of pixels of gray value in each range was counted to react the fluid volume. From the X-CT scan of Figure 13, it can be seen that the density of fluid is smaller than that of a rock skeleton, and the gray value is low. Each gray data point was read and divided, and the number of pixels was calculated. The statistical data are shown in Figure 14.

For two types of reservoirs, when the gray value is 40, it indicates the density gray value of gas phase in porous media. The number of pixel points shows a decreasing trend from top to bottom of the core. There is a high volume of gas phase in the upper part of the core, where the oil is replaced by a surfactant. In the oil phase gray value area of 60–140, the number of pixels in the position of 0–24 mm of type I core is less and uniform, and the oil phase is effectively stripped. When the position is greater than 24 mm, the number of pixels increases significantly, and there is more oil content here. The high gray range (greater than 140) shows the opposite trend. The content of high-density phase is relatively high at the position of 24–30 mm, and the volume of rock skeleton accounts for a large proportion. In contrast, the volume proportion of low-density phase in type II reservoir is low and the distance of oil phase discharge is closer, only in the range of 8–10 mm.

## 4 Conclusions

This paper describes the oil stripping efficiency of the surfactant and the mechanism of surfactant imbibition in porous media during the development of tight reservoirs. High oil stripping efficiency can be achieved when the interfacial tension is below 2 mN/m, the surface tension is 25–32 mN/m, and the wetting angle is between 10° and 22°. Due to the existence of surfactant adsorption probability, the adhesion work required for high stripping efficiency is 49–62 mN/m, and the corresponding stripping efficiency can reach 46.73–70.56%. The demulsification rate of emulsion has no obvious correlation with the stripping efficiency, while the average particle size of emulsion can effectively characterize the correlation between particle size and stripping efficiency. In the process of tight core imbibition, there are two main ways of imbibition. One is emulsification stripping and thermal diffusion–convection. The majority of the surfactant molecules combine with the oil to form emulsion particles, allowing the stripping of the oil from the porous medium. In the second type of imbibition, surfactants make the core surface more water-wet, and as the aqueous solution continues to penetrate deeper into the pore medium, capillary forces are able to achieve the effect of oil displacement. The present research can enrich the study of enhanced oil recovery in tight reservoirs.
